# Complete genome sequences of six *Basfia succiniciproducens* isolates, a biotechnologically relevant species

**DOI:** 10.1128/mra.00945-25

**Published:** 2025-09-24

**Authors:** Peter Kuhnert, Jing Zhang

**Affiliations:** 1 Institute of Veterinary Bacteriology, Vetsuisse Faculty, University of Bern 27210, Bern, Switzerland; Wellesley College, Wellesley, Massachusetts, USA

**Keywords:** biotechnology, succinic acid, bovine rumen, sustainability, taxonomy

## Abstract

Complete circular genomes of six *Basfia succiniciproducens* strains are reported. The sequences provide insight into the biotechnological potential this species has for the sustainable bio-production of succinic acid as an alternative basic component for currently fossil-based counterparts in chemical industries.

## ANNOUNCEMENT

*Basfia succiniciproducens* is a key part of the bovine rumen microbiome (1). *B. succiniciproducens* is also the preferred and validly published name for *"Mannheimia succiniciproducens*" often found in the literature. As its name indicates, it has the potential to produce succinic acid from sugars and glycerol (2, 3). It shares this characteristic with other biotechnologically interesting bacteria, for example, *Actinobacillus succinogenes,* another member of the *Pasteurellaceae* family (4, 5). Succinic acid is a key chemical building block, and its biotechnological synthesis has become more attention in the light of sustainability since it can replace petrochemical-based production (6–8).

Six strains of *B. succiniciproducens*, including the type strain, were isolated from freshly collected bovine rumen juice using a *Pasteurellaceae* selective medium (PSM) (9). A 50 µL aliquot of rumen juice diluted 1:10 in Luria-Bertani medium was streaked on PSM agar plates and incubated overnight at 37°C and 5% CO_2_. Single colonies identified by 16S rRNA gene sequencing as *B. succiniciproducens* were subcultured on Trypticase Soy Agar plates (TSA; Becton Dickinson) with 5% sheep blood at 37°C and 5% CO_2_ for 24 h. Strains were kept for long-term storage at −70°C and were freshly grown for genome sequencing on TSA blood agar plates. Genomic DNA was isolated from a few colonies of freshly grown strains JF4016^T^, JF4134, JF4136, JF4141, JF4142, and JF4213 using phenol-chloroform extraction and isopropanol precipitation (10) followed by purification with the DNeasy PowerClean Cleanup Kit (Qiagen).

To assess DNA quality and quantity, Fragment Analyzer (Advanced Analytical Technologies) and Qubit fluorometry (Thermo Fisher Scientific) were used, respectively. DNA shearing was achieved by tagmentation. Fragments < 3 kb were then removed based on the LongPlex Long Fragment Multiplexing Kit protocol (seqWell) using 35% diluted AMPure beads. Libraries were prepared using the LongPlex Kit and SMRTbell Prep Kit 3.0 (Pacific Biosciences) and profiled on a Fragment Analyzer. Sequencing was performed on a PacBio Revio system (1 SMRT Cell 25M, 30-hour movie time, Revio chemistry).

Adapter trimming and demultiplexing were accomplished using SMRT Link v13.0.1, followed by additional adapter removal with LongPlex Demultiplex Nextflow (seqWell) on the Lausanne sequencing platform. NanoPlot v1.44.1 (11) was used to assess read quality. Genome assemblies were generated using Flye v2.9.4-b1799 (12, 13). Assembly quality was evaluated using QUAST v5.3.0 (14) and BUSCO v5.8.2 (15). Circular genomes were rotated to start at *dnaA* using Circlator v1.5.5 (16). The annotation was added by the NCBI Prokaryotic Genome Annotation Pipeline (PGAP; version 6.10). Default parameters were used for all software.

Genome statistics of strains are summarized in Table 1.

**TABLE 1:** Genome assembly statistics and accession numbers

Strain						
	JF4016^T^	JF4134	JF4136	JF4141	JF4142	JF4213
Total # of reads	116,577	132,519	166,623	156,057	137,071	89,321
Read *N*₅₀ (bp)	12,000	12,054	11,332	11,349	11,847	11,370
Genome size	2,268,443	2,287,598	2,320,518	2,354,985	2,322,668	2,329,372
Coverage	549	626	735	679	625	390
Assembly *N*₅₀ (bp)	2,268,443	2,287,598	2,320,518	2,354,985	2,322,668	2,329,372
BUSCO scores	99.1	98.9	99.2	99.1	99.0	99.2
GC%	42.44	42.49	42.42	42.48	42.44	42.46
CDS (protein)	2,020	2,071	2,090	2,144	2,117	2,106
tRNA	60	60	60	60	60	60
rRNA (SS, 16S, 23S)	7, 6, 6	7, 6, 6	7, 6, 6	7, 6, 6	7, 6, 6	7, 6, 6
GenBank Acc. No.	CP197716	CP197715	CP197714	CP197713	CP197712	CP197711

## Data Availability

The complete genome sequences of strains JF4016^T^, JF4134, JF4136, JF4141, JF4142 and JF4213 have been deposited in GenBank under the accession numbers CP197716, CP197715, CP197714, CP197713, CP197712, and CP197711, respectively. The associated BioSamples are SAMN49888341, SAMN49888342, SAMN49888343, SAMN49888344, SAMN49888345, and SAMN49888346, respectively. The raw reads were deposited under accession numbers SRR34920848, SRR34920847, SRR34920846, SRR34920845, SRR34920844, and SRR34920843, respectively. All data is contained in BioProject PRJNA1289394.
